# Supplementary material for: The Complete Mitochondrial Genome of One Breeding Strain of Asian Swamp Eel (Monopterus albus, Zuiew 1793) Using PacBio and Illumina Sequencing Technologies and Phylogenetic Analysis in Synbranchiformes
Source: Genes (Basel). 2021 Oct 1;12(10):1567. doi: 10.3390/genes12101567 (PMC8535454; doi:10.3390/genes12101567)
Supplement: Supplementary file 1 [file genes-12-01567-s001.zip › Figure S1.pdf]

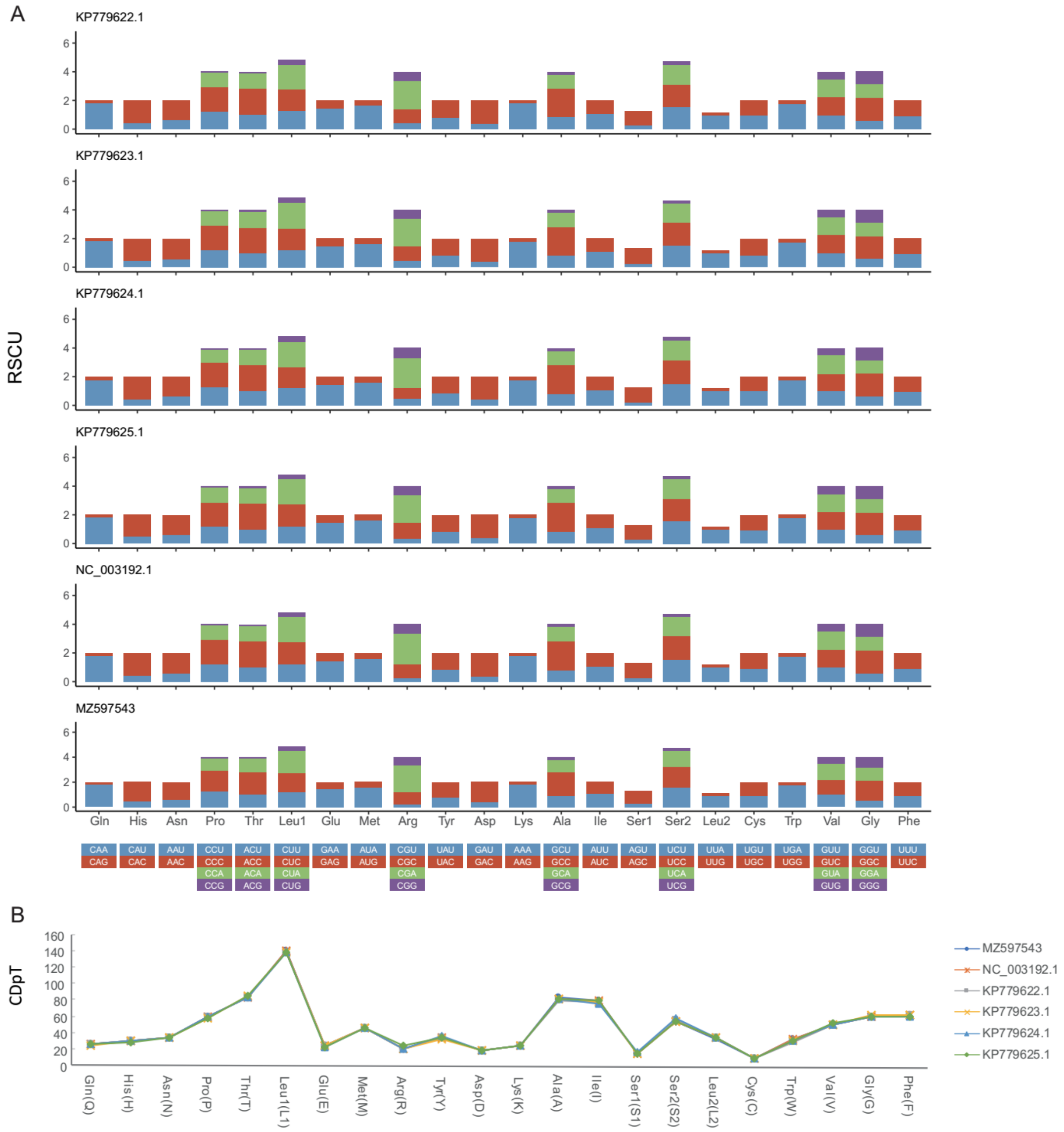

Figure S1: Codon frequency and RSCU (relative synonymous codon usage) in the mitogenomes of selecting breed, and the other five ones of *M. albus* sampled from different sites.
