# Supplementary material for: The Complete Mitochondrial Genome of One Breeding Strain of Asian Swamp Eel (Monopterus albus, Zuiew 1793) Using PacBio and Illumina Sequencing Technologies and Phylogenetic Analysis in Synbranchiformes
Source: Genes (Basel). 2021 Oct 1;12(10):1567. doi: 10.3390/genes12101567 (PMC8535454; doi:10.3390/genes12101567)
Supplement: Supplementary file 1 [file genes-12-01567-s001.zip › Figure S2.pdf]

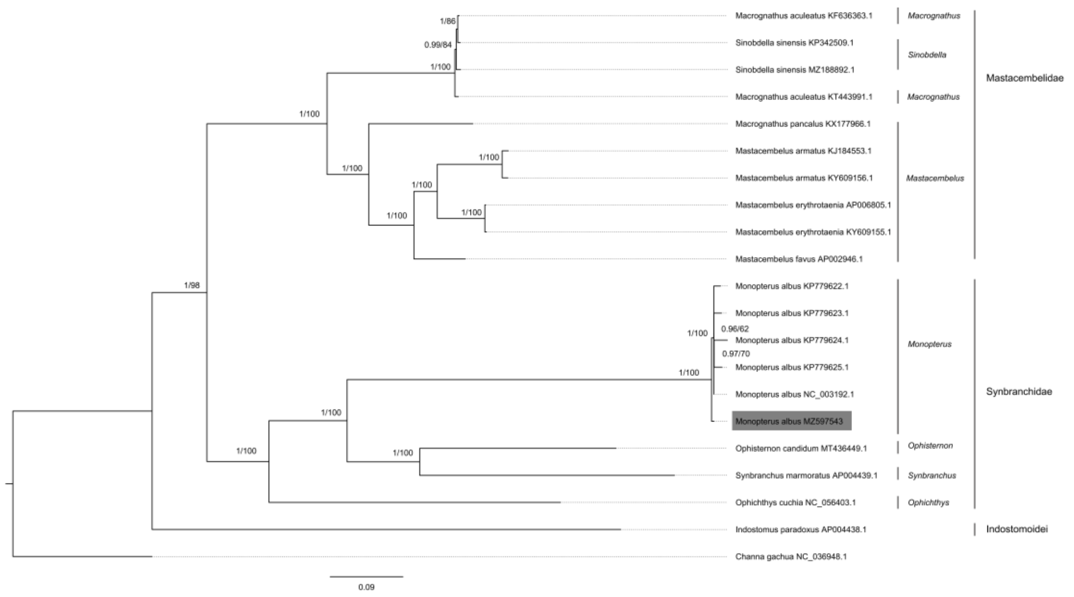

Figure S2: The phylogenetic tree of order Synbranchiformes inferred from the concatenated 13 PCGs, 2 rRNAs, 22 tRNAs and one D-loop of 20 complete mitochondrial sequence obtained using MrBayes v.3.2.6 and IQ-TREE v.1.5.5. *Channa gachua* (NC_036948.1) was used as outgroup.
