# Supplementary material for: The Complete Mitochondrial Genome of One Breeding Strain of Asian Swamp Eel (Monopterus albus, Zuiew 1793) Using PacBio and Illumina Sequencing Technologies and Phylogenetic Analysis in Synbranchiformes
Source: Genes (Basel). 2021 Oct 1;12(10):1567. doi: 10.3390/genes12101567 (PMC8535454; doi:10.3390/genes12101567)
Supplement: Supplementary file 1 [file genes-12-01567-s001.zip › Figure S3.pdf]

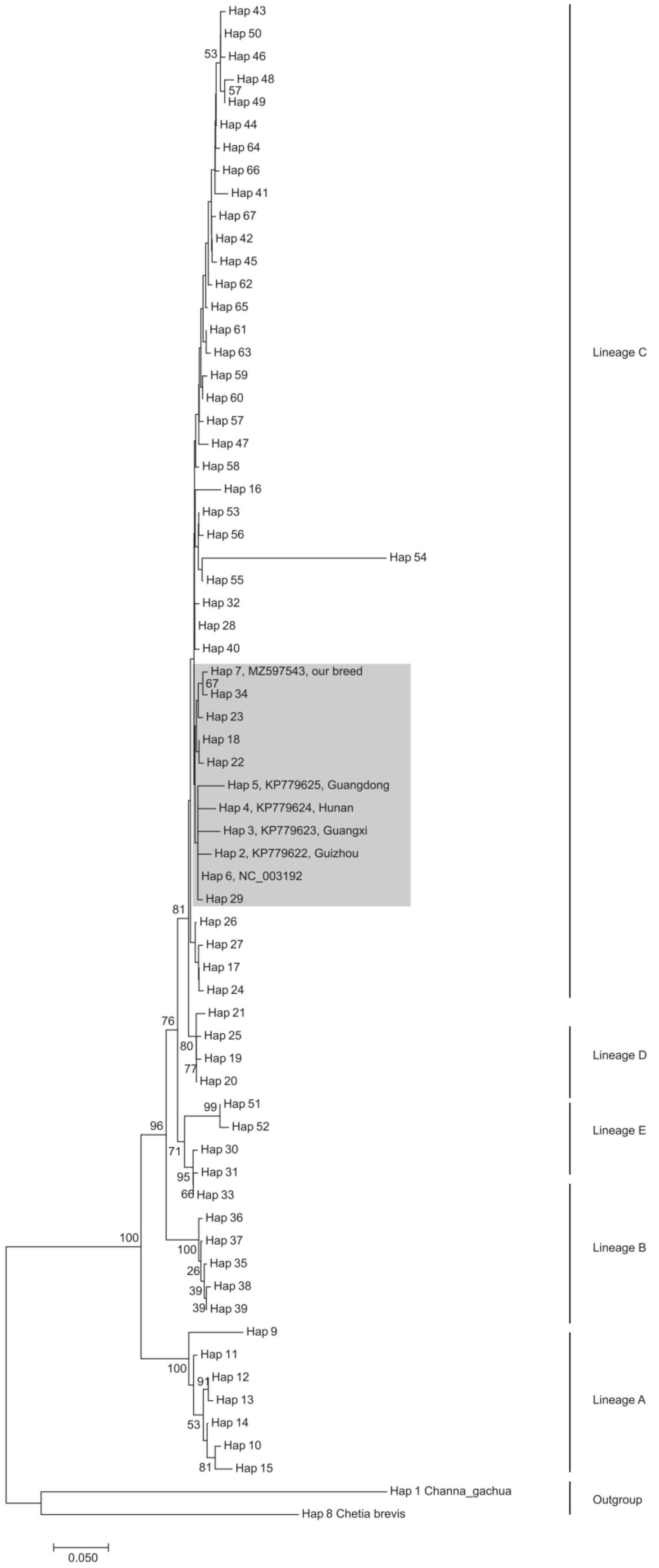

Hap 43
Hap 50
Hap 46
Hap 48
Hap 49
Hap 44
Hap 64
Hap 66
Hap 41
Hap 67
Hap 42
Hap 45
Hap 62
Hap 65
Hap 61
Hap 63
Hap 59
Hap 60
Hap 57
Hap 47
Hap 58
Hap 16
Hap 53
Hap 56
Hap 54
Hap 55
Hap 32
Hap 28
Hap 40
Hap 7, MZ597543, our breed
Hap 34
Hap 23
Hap 18
Hap 22
Hap 5, KP779625, Guangdong
Hap 4, KP779624, Hunan
Hap 3, KP779623, Guangxi
Hap 2, KP779622, Guizhou
Hap 6, NC_003192
Hap 29
Hap 26
Hap 27
Hap 17
Hap 24
Hap 21
Hap 25
Hap 19
Hap 20
Hap 51
Hap 52
Hap 30
Hap 31
Hap 33
Hap 36
Hap 37
Hap 35
Hap 38
Hap 39
Hap 9
Hap 11
Hap 12
Hap 13
Hap 14
Hap 10
Hap 15
Hap 1 Channa_gachua
Hap 8 Chetia brevis
Lineage C
Lineage D
Lineage E
Lineage B
Lineage A
Outgroup
0.050

Figure S3: The neighbour-joining tree of the phylogenetic relationships among 67 haplotypes of D-loop of our breed (MZ597543) and wild swamp eel species from China based on p-distances. Table S1: Nucleotide composition and skewness levels calculated for sequenced majority strand of the mitogenomes of *M. albus*.
