# Supplementary material for: The Complete Mitochondrial Genome of One Breeding Strain of Asian Swamp Eel (Monopterus albus, Zuiew 1793) Using PacBio and Illumina Sequencing Technologies and Phylogenetic Analysis in Synbranchiformes
Source: Genes (Basel). 2021 Oct 1;12(10):1567. doi: 10.3390/genes12101567 (PMC8535454; doi:10.3390/genes12101567)
Supplement: Supplementary file 1 [file genes-12-01567-s001.zip › Table S1.pdf]

Table S1. Nucleotide composition and skewness levels calculated for sequenced majority strand of the six mitogenomes of *Monopterus albus*

**Region(s)/Genes**	**Size(bp)**	**Nucleotides Composition**						**AT-Skew**	**GC-Skew**
		**A**	**T**	**G**	**C**	**A+T**	**G+C**		
MZ597543, selecting breed									
Whole mitogenome	16621	28.9	27.1	14.5	29.5	56	44	0.032	-0.341
PCGs	10899	26.5	28.4	13.5	31.6	54.9	45.1	-0.034	-0.403
1st condon positions	3633	28.5	21.4	22.2	27.9	49.9	50.1	0.141	-0.113
2nd condon positions	3633	28.1	40.3	18.9	12.7	59.2	40.8	-0.362	-0.379
3rd condon positions	3633	32.2	23.5	5.5	38.9	55.7	44.4	0.157	-0.753
tRNAs	983	32	27.1	18.1	22.8	59.1	40.9	0.084	-0.114
rRNAs	2621	34.4	22.6	18.6	24.4	57	43	0.206	-0.136
KP779622.1, Guizhou province									
Whole mitogenome	16622	28.8	27.2	14.5	29.5	56	44	0.029	-0.342
PCGs	10902	26.5	28.5	13.4	31.6	55	45	-0.038	-0.405
1st condon positions	3634	28.5	21.6	22	27.9	50.1	49.9	0.137	-0.12
2nd condon positions	3634	19.1	40.1	12.6	28.2	59.2	40.8	-0.355	-0.382
3rd condon positions	3634	31.8	23.9	5.6	38.7	55.7	44.3	0.142	-0.748
tRNAs	985	32.3	27	18	22.7	59.3	40.7	0.089	-0.117
rRNAs	2625	34.4	22.6	18.5	24.5	57	43	0.206	-0.138
KP779623.1, Guangxi province									
Whole mitogenome	16622	28.9	27.1	14.5	29.4	56	43.9	0.032	-0.339
PCGs	10902	26.6	28.4	13.5	31.4	55	44.9	-0.033	-0.398
1st condon positions	3634	28.7	21.5	22.2	27.6	50.2	49.8	0.145	-0.107
2nd condon positions	3634	19	40.3	12.7	28	59.3	40.7	-0.36	-0.376
3rd condon positions	3634	32.1	23.4	5.7	38.7	55.5	44.4	0.156	-0.743
tRNAs	983	32.1	27.1	18	22.8	59.2	40.8	0.086	-0.117
rRNAs	2622	34.3	22.6	18.3	24.8	56.9	43.1	0.206	-0.15
KP779624.1, Hunan province									
Full genome	16622	28.8	27.2	14.5	29.5	56	44	0.028	-0.342
PCGs	10902	26.4	28.6	13.5	31.6	55	45.1	-0.04	-0.402
1st codon position	3634	28.2	22	21.9	27.9	50.2	49.8	0.124	-0.121

2nd codon position	3634	19.1	40	12.6	28.4	59.1	41	-0.354	-0.386
3rd codon position	3634	31.9	23.8	6	38.4	55.7	44.4	0.146	-0.731
tRNAs	983	32	27.2	18	22.8	59.2	40.8	0.082	-0.117
rRNAs	2622	34.5	22.6	18.3	24.6	57.1	42.9	0.209	-0.146
KP779625.1, Guangdong province									
Full genome	16622	28.9	27.2	14.6	29.4	56.1	44	0.03	-0.337
PCGs	10902	26.5	28.5	13.6	31.4	55	45	-0.037	-0.396
1st codon position	3634	28.6	21.5	22.1	27.8	50.1	49.9	0.143	-0.116
2nd codon position	3634	18.9	40.2	12.9	28	59.1	40.9	-0.361	-0.368
3rd codon position	3634	32	23.8	5.8	38.4	55.8	44.2	0.146	-0.74
tRNAs	983	32.3	26.9	18	22.8	59.2	40.8	0.093	-0.117
rRNAs	2622	34.4	22.6	18.5	24.6	57	43.1	0.207	-0.141
NC_003192.1, unknown									
Full genome	16622	28.9	27.2	14.5	29.5	56.1	44	0.031	-0.341
PCGs	10899	26.5	28.5	13.4	31.6	55	45	-0.036	-0.403
1st codon position	3633	28.5	21.5	22.2	27.8	50	50	0.139	-0.111
2nd codon position	3633	18.9	40.3	12.7	28.1	59.2	40.8	-0.362	-0.378
3rd codon position	3633	32.1	23.6	5.4	38.8	55.7	44.2	0.153	-0.755
tRNAs	983	32.1	27.1	18	22.8	59.2	40.8	0.086	-0.117
rRNAs	2622	34.4	22.5	18.5	24.5	56.9	43	0.209	-0.14
